# On the Formation of Tubercles in Various Organs

**Published:** 1839-10-05

**Authors:** W. W. Gerhard


### CLINICAL LECTURE.

### ON TUBERCULOUS DISEASE.

LECTURE III.

*On the formation of Tubercles in various organs.*

By W. W. GERHARD, M. D.

TUBERCLES are not deposited throughout all the tissues of the body in an equal degree of frequency. The relative tendency of the disease to particular situations, is influenced by age, sex, and perhaps by some other causes of a less appreciable nature. Various estimates have been made by different observers as to the relative frequency of tubercles; the following are the conclusions to which we have arrived. Taking all ages together, tubercles are vastly more frequent in the lungs than in any other organ of the body. Next in order is the glandular or lymphatic system, particularly the bronchial, the mesenteric, and the cervical glands. The deposits of tubercles in the lymphatic system occur chiefly amongst children, and are most frequent in the bronchial glands; in adults the same deposit is quite common, but it is much more frequent in the mesenteric glands. The tuberculous affection of the lungs, although common in children, is rather less frequent than the tuberculous lesion of the bronchial glands; in adults the reverse of this proposition holds good. Hence we may say, in general terms, that the tuberculous disorder in most cases first shows itself in the lungs, and next in frequency attacks the glandular system.

After the lungs and lymphatic glands, which, above all other tissues of the body, are the chosen seat of tubercles, we must place the large intestine, then the serous membranes, in the order of pleuræ, peritoneum, and arachnoid, (rarely the pericardium,) then the spleen, and afterwards the kidney, liver, and various parts of the osseous system. The organs which are more rarely the seat of tubercle than those we have just enumerated, cannot, of course, be so readily classed in a regular series as those tissues which are very commonly the seat of this morbid product.

You will find the classification of tubercles of the various organs which is given by Andral, in his Pathological Anatomy, a very good one; it is, however, not strictly correct in all respects. He has not laid sufficient stress upon the tuberculous disease of the serous membranes, or of the bones. This omission arises in part from the gradual progress of our knowledge of pathological anatomy, which is quite observable since the publications of Andral's work, &c., in part from the class of patients observed by M. Andral, who were almost exclusively patients in the medical wards of a Parisian hospital, and not taken at hazard from both medical and surgical wards. The classification of the relative frequency of tubercles you will find in Andral's Pathological Anatomy. I quote from Townsend's translation, p. 422, vol. i.

Of the three hundred and fifty individuals whom he examined with tuberculous disease, M. Louis found tubercles in the lungs in every case but one, in which there were tubercles in any organ of the body. Besides the lungs, these three hundred and fifty subjects offered tubercles in the other organs, in the following proportions:

| | | |
|---|---|---|
| In the small intestine, | in one-third of the bodies. | |
| In the large intestine, | one-ninth | " |
| In the mesenteric glands, | one-fourth | " |
| In the cervical glands, | one-tenth | " |
| In the lumbar glands, | one-twelfth | " |
| In the prostate, | one-thirteenth | " |
| In the spleen, | one-fourteenth | " |
| In the ovaries, | one-twentieth | " |
| In the kidneys, | one-fortieth | " |
| In the uterus, | in one case. | |
| In the cerebrum, | do. | |
| In the cerebellum, | do. | |
| In the ureter, | do. | |

M. Lombard's calculations, which I also quote from Andral's Pathological Anatomy, agree nearly with those of Dr. Louis, except that the organs which contain tubercles at the same time that the lungs are affected, are not attacked in so great a proportion as in the subjects examined by Dr. Louis. The latter observer is probably the more correct. There is no doubt that tubercles were actually present in every organ mentioned by Dr. Louis, while the negative evidence which causes Dr. Lombard to regard the proportionate frequency of the tuberculous deposit in certain organs, such as the spleen and small intestine, as less considerable than the ratio stated by Dr. Louis, is, from its very nature, liable to error. Dr. Lombard, however, has extended his observations to a larger number of organs,—amongst others, to the sub-arachnoid tissue and the false membrane of the pleura. In these portions of the body tuberculous deposits are vastly more frequent than would seem indicated by the calculation of M. Lombard, who states the proportion of cases in which he found tubercles in the false membranes to be only two in a hundred cases. This must certainly be an error,—for the serous tissue and its false membranes are undoubtedly one of the most frequent seats of tuberculous disease, and not amongst the rare complications of these disorders.

The error, or rather the defect of observation, must have arisen from the comparative difficulty of inspecting the condition of the serous membranes, which are more concealed than the parenchymatous organs.

Dr. Lombard has also made an estimate of the relative frequency of tubercles in various portions of the body in children. The following table is drawn up by Dr. Lombard. In one hundred bodies of tuberculous children Dr. L. found tubercles in the lungs seventy-three times, of which thirty times in one single lung, thirteen times in the left, and seventeen times in the right lung.

| | |
|---|---|
| In the bronchial glands, . . | 87 times. |
| In the mesenteric glands, . . | 31 " |
| In the spleen, . . . . . | 25 " |
| In the kidneys, . . . . . | 11 " |
| In the intestines, . . . | 9 " |
| In the nervous centres, . . | 9 " |
| In the cervical glands, . . | 7 " |
| In the meninges, . . . | 6 " |
| In the pancreas, . . . . . | 5 " |
| In the gastro-hepatic glands, . | 5 " |
| In the sub-peritoneal cellular tissue, | 5 " |
| In the spleen, . . . . . | 4 " |
| In the inguinal glands, . . | 3 " |
| In the sub-pleuritic cellular tissue, | 2 " |
| In the lumbar glands, . . . | 1 " |
| In the sub-mucous tissue of the bladder, | 1 " |
| In the epiploon, . . . . . | 1 " |
| In the coats of the gall-bladder, . | 1 " |
| In the false membranes of the bladder, | 1 " |

You perceive, therefore, as I have previously stated, that the proportion of tuberculous deposits in the different organs of the body, varies greatly in children and adults; and that the chief points of difference are to be found in the extreme tendency of children to the tuberculous diseases of the glandular system. My own observations agree essentially with those of Dr. Lombard. I should, however, place the proportion of tubercles in the bronchial glands still higher; for, in many cases, although these bodies do not exist at the moment, we have the evidence of cicatrices or of calcareous concretions to prove that the deposit of tubercle formerly occurred, but has been removed by the usual process of absorption.

There is some difference as to the connection of tubercle in various points with the general diathesis giving rise to the disorder. Thus, it would seem that pulmonary phthisis is the result of a more intense degree of tuberculous cachexia than the same lesion of the bronchial glands, and is more apt to be followed by emaciation and hectic fever. This, however, is a matter for future examination.

After pointing out to you the seat and mode of development of tubercle, we must inquire if the disease tends to pass through the same course in various parts of the body. A superficial observer who confines himself to the symptoms alone, will unhesitatingly tell you that this is not the case. But when we carefully examine into the matter, we find that the difference does not depend upon a peculiarity of the disease, anatomically considered, but purely on the difference of tissue in which it is seated, which either retards or promotes the growth of tubercle, and gives rise to the symptoms of general tuberculous disease, which are modified by the alteration which may take place in the functions of the organ in which the tubercle is seated.

At this stage of our inquiry we are naturally led to examine the general symptoms connected with the formation and progress of tubercle, and ascertain, if possible, whether there are symptoms of the disease which are independent of the local functional disturbance. For, in the great majority of cases, the local symptoms which occur in the early stages of tubercle, depend either upon the inflammation which may chance to attend its secretion, or upon the reaction excited in the tissue by the presence of an anomalous tissue, which may be considered in most respects a foreign body, and therefore as an irritant.

If we examine the cases of chronic tuberculous disease, it is very evident that the symptoms must often be extremely obscure, because the nature of the disease is to advance so slowly that the system becomes, as it were, habituated to the new growth of morbid structure, and betrays the disease by few signs of reaction against the diseased tendency. Hence we must first seek for the signs of general tuberculous disease in the more acute cases, and afterwards pass to those which are both more chronic and more obscure.

